# Comparative analysis of virulence and resistance gene profiles between carbapenem-resistant and ESBL-producing *Escherichia coli*

**DOI:** 10.3389/fmicb.2026.1843914

**Published:** 2026-07-15

**Authors:** Dandan Lou, Linhui Huang, Yanzi Chang, Xudong Feng, Hui Gao, Hong Li, Yanye Tu

**Affiliations:** 1 Department of Clinical Laboratory, The Affiliated Lihuili Hospital of Ningbo University, Ningbo, China; 2 Health Science Center, Ningbo University, Ningbo, Zhejiang, China

**Keywords:** carbapenemase, *Escherichia coli*, extended-spectrum β-lactamase, resistance gene, virulence gene

## Abstract

**Objective:**

This study aimed to compare the virulence characteristics and antimicrobial resistance gene profiles of extended-spectrum β-lactamase-producing *Escherichia coli* (ESBL-EC) and carbapenem-resistant *Escherichia coli* (CR-EC), and to explore the potential effects of these traits on the evolutionary dynamics of *Escherichia coli* and the prevention and control of healthcare-associated infections in Ningbo.

**Methods:**

A total of 112 *E. coli* isolates obtained from The Affiliated Lihuili Hospital of Ningbo University between January 2021 and December 2023 were included, comprising 72 ESBLs-EC isolates and 40 CR-EC isolates. Bacterial identification and antimicrobial susceptibility testing were performed with the VITEK-2 Compact system, and all susceptibility results were verified by the Kirby–Bauer disk diffusion method. Polymerase chain reaction (PCR) was applied to detect virulence genes and resistance genes. Clonal lineage analysis of the 112 *E. coli* isolates was performed with GrapeTree software.

**Results:**

Among the 112 *E. coli* isolates subjected to sequence type (ST) analysis, ST131 was the most common ST among ESBLs-EC isolates, whereas ST410 accounted for the largest proportion among CR-EC isolates. PCR-based screening of resistance genes showed that *bla*_*CTX–M*_ was the predominant β-lactam resistance gene carried by ESBLs-EC isolates (93.06%, 67/72), whereas *bla*_*TEM*_ was predominant in CR-EC isolates (52.50%, 21/40), with a statistically significant difference between the 2 groups (*P* < 0.05). No carbapenemase resistance genes were detected in ESBLs-EC isolates, whereas *bla*_*NDM*_ and *bla*_*KPC*_ were detected in 62.50% (25/40) and 7.50% (3/40) of CR-EC isolates, respectively, with significant differences between the 2 groups (*P* < 0.05). PCR-based virulence gene typing showed that, after Bonferroni correction, the carriage proportions of the iron acquisition system genes *fyuA* (72.22% vs. 40.00%) and *iutA* (69.44% vs. 37.50%) and the PAI (40.28% vs. 0%) were significantly higher in ESBLs-EC isolates than in CR-EC isolates, with all corrected *P* values < 0.05.

**Conclusion:**

At present, *E. coli* isolates circulating in Ningbo are mainly represented by *bla*_*CTX–M*_-type ESBLs-EC and *bla*_*NDM*_-type CR-EC, showing an evolutionary trend characterized by high resistance and low virulence.

## Introduction

*Escherichia coli*, one of the most common opportunistic pathogens in clinical settings, is widely distributed in the human intestinal tract and natural environments. In immunocompromised populations, *E. coli* can cause a range of severe infectious diseases, including urinary tract infection, intra-abdominal infection, and bloodstream infection (Ilmavirta et al., 2023). It is also an important pathogen involved in hospital-acquired and community-acquired infections, posing a substantial threat to public health. The inappropriate use of antimicrobial agents in clinical practice has contributed to the development of antimicrobial resistance in *E. coli* (Quan et al., 2021; Yang et al., 2022). As resistance levels continue to rise, *E. coli* has become a major challenge for global health care systems (Huang et al., 2024).

Among the resistance mechanisms of *E. coli*, the production of extended-spectrum β-lactamases (ESBLs) is one of the major causes of resistance to β-lactam antimicrobial agents, including penicillins and cephalosporins, mainly third-generation cephalosporins. ESBLs hydrolyze the β-lactam ring of these antimicrobial agents, thereby abolishing their antibacterial activity (Wein et al., 2020). Common ESBL genes detected in clinical settings include the CTX-M, TEM, and SHV types, among which CTX-M has become the dominant genotype in ESBL-producing *E. coli* (ESBLs-EC) worldwide, given its wide dissemination and high prevalence (Rios et al., 2022; Musicha et al., 2026). With the spread of ESBLs-EC, carbapenems, such as imipenem and meropenem, are often required for the treatment of infections caused by these isolates. Owing to their broad antibacterial spectrum and strong antibacterial activity, these agents were once regarded as one of the last-line therapeutic options for multidrug-resistant (MDR) Gram-negative infections (Macesic et al., 2025).

However, extensive clinical exposure to carbapenems has been accompanied by the emergence of carbapenem-resistant E. coli (CR-EC). CR-EC mainly acquires resistance to carbapenem antibiotics through the production of carbapenemases, including KPC, NDM, OXA-48, VIM, and IMP enzymes, which hydrolyze carbapenems (Li et al., 2024a). Infections caused by carbapenem-resistant Enterobacterales (CRE) have severely restricted clinical treatment options and have been listed by the World Health Organization (WHO) as priority pathogens requiring new antimicrobial agents (Li et al., 2024b; Yeoh et al., 2025). Their dissemination has also become a threat to global public health security (Das et al., 2023).

Current clinical studies on ESBLs-EC and CR-EC have largely centered on resistance phenotype testing, genotype distribution, or epidemiological characteristics of single isolate groups. Comparative data remain limited on whether these 2 categories of *E. coli* with different resistance levels, namely ESBL-producing isolates that remain susceptible to carbapenems and CR-EC isolates that are resistant to carbapenems, differ in virulence characteristics, including adhesion capacity, invasive ability, biofilm-forming capacity, and virulence gene carriage, or in the composition of their resistance gene profiles. Defining the differences in virulence and resistance gene profiles between these 2 groups may help clarify the potential relationship between bacterial resistance and virulence, such as whether acquisition of resistance genes is accompanied by increased or decreased virulence, and may also provide evidence for optimizing clinical treatment strategies. Therefore, this study included clinically isolated ESBLs-EC and CR-EC strains as the study objects and compared the virulence characteristics and resistance gene profiles of the 2 groups. The aim was to examine the evolutionary trends of resistance and virulence in *E. coli* in Ningbo, expand the molecular epidemiological characterization of *E. coli* with different resistance phenotypes, clarify its evolutionary patterns, and provide a reference for precision antimicrobial therapy in clinical practice.

## Materials and methods

### Bacterial isolate sources

A total of 112 non-duplicate *E. coli* isolates were collected from The Affiliated Lihuili Hospital of Ningbo University between January 2021 and December 2023, including 72 ESBLs-EC isolates and 40 CR-EC isolates. The specimen types included urine, whole blood, sputum, feces, drainage fluid, and others. Duplicate isolates recovered from the same anatomical site of the same patient were excluded with WHONET 5.6 software.

### Instruments and reagents

The VITEK-2 Compact automated microbial identification system was obtained from bioMérieux, France. Imipenem, ceftazidime and other antimicrobial disks were purchased from Oxoid, United Kingdom (batch number: 3376197). Susceptible (S), intermediate (I), and resistant (R) results for routine clinical antimicrobial agents were interpreted according to Clinical and Laboratory Standards Institute (CLSI) guidelines. Primer synthesis and PCR product processing were completed by Sangon Biotech Co., Ltd., Shanghai, China.

### Methods

#### Bacterial identification and antimicrobial susceptibility testing

The collected isolates were inoculated onto Columbia blood agar plates and incubated overnight at 37°C. A single colony was selected for bacterial identification and in *vitro* antimicrobial susceptibility testing with the VITEK-2 Compact automated microbial identification system (bioMérieux, France). All susceptibility results were verified by the Kirby–Bauer disk diffusion method.

Susceptibility breakpoints for clinical antimicrobial agents were interpreted as S, I, or R according to the CLSI M100 standard. The quality-control strain for antimicrobial susceptibility testing, E. coli ATCC 25922, was obtained from the bacterial strain repository of our hospital.

#### Confirmatory testing for ESBLs and CRE

Confirmatory testing for ESBLs was performed according to CLSI guidelines with ceftazidime and ceftazidime/clavulanic acid disks, as well as cefotaxime and cefotaxime/clavulanic acid disks. An increase in the inhibition zone diameter of ≥ 5 mm for disks containing clavulanic acid, relative to the corresponding disks without clavulanic acid, was defined as ESBLs production. Confirmatory testing for CRE was performed with the modified Hodge test according to CLSI recommendations. Detection of carbapenem resistance was based on the CLSI M100 (Pierce et al., 2023) standard, under which isolates with a minimum inhibitory concentration (MIC) ≥ 4 μg/mL for imipenem or meropenem were defined as CR-EC.

#### DNA template extraction

DNA templates of *E. coli* were extracted by high-temperature lysis. Autoclaved EP tubes were filled with 0.2 mL of distilled water. A single fresh colony was picked with a sterile cotton swab and transferred onto the inner wall of the EP tube. The tube was heated in a metal bath at 100°C for 10 min, followed by high-speed centrifugation at 2,000 rpm. The supernatant was transferred to a fresh EP tube and stored at -20°C until further analysis.

#### Multilocus sequence typing

According to the Achtman scheme (Wirth et al., 2006), 7 housekeeping genes, including *adk*, *fumC*, *gyrB*, *icd*, *mdh*, *purA*, and *recA*, were amplified, and the amplification products were sent to Sangon Biotech, Shanghai, for sequencing. The obtained sequences were submitted to the MLST database^1^ for comparison, and the allele number of each housekeeping gene was assigned to determine the MLST type of each isolate. GrapeTree software was applied to analyze clonal relationships among the *E. coli* isolates.

PCR was performed in a 50 μL reaction system containing 2 μL each of upstream and downstream primers at 10 μmol/L, 2.5 μL of dNTPs at 2 mmol/L, 5.0 μL of PCR buffer containing Mgcl2, 0.5 μL of La Taq DNA polymerase at 5 U/μL, ddH_2_O added to 49 μL, and 1.0 μL of DNA template. The PCR conditions were as follows: initial denaturation at 95°C for 4 min; denaturation at 94°C for 30 s, annealing for 45 s, and extension at 72 °C for 1 min for 30 cycles; and final extension at 72°C for 7 min. A 5 μL aliquot of the PCR product was subjected to electrophoresis on a 1.5% agarose gel (110 V, 20 min). Primer sequences and reaction conditions are provided in Supplementary Material 1.

#### Detection of virulence genes

Virulence genes were detected by PCR, including ➀ adhesion-associated genes, *fimH*, *papA*, *papC*, *Sfa*, and *focG*; ➁ iron acquisition system genes, *iroN, fyuA*, *sitA*, and *iutA;* ➂ toxin-associated genes, *hlyA*, *cnf1*, and *cvaC*; ➃ serum resistance and capsule-associated genes, *rfc*, *traT*, *kpsMT II*, *kpsMT K1*, and *kpsMT K5*; and ➄ the pathogenicity island gene (PAI). Primer sequences and reaction conditions are provided in Supplementary Material 1.

The PCR reaction system contained 1 μL of DNA template, 12.5 μL of Taq enzyme, 1 μL each of upstream and downstream primers, and 9.5 μL of ddH_2_O, yielding a total reaction volume of 25 μL. The reaction conditions were as follows: initial denaturation at 94 °C for 5 min; denaturation at 94°C for 30 s, annealing at 56 °C for 45 s, and extension at 72°C for 30 s for 25 cycles; and final extension at 72°C for 5 min. A 5 μL aliquot of the PCR product was subjected to electrophoresis on a 1.5% agarose gel (110 V, 20 min).

#### Detection of resistance genes

PCR was applied to detect β-lactam resistance genes, including *bla*_*CTX–M*_*, bla*_*SHV*,_ and *bla*_*TEM*,_ as well as carbapenemase genes, including *bla*_*KPC*_, *bla*_*NDM*_, *bla*_*VIM*_, *bla*_*IMP*_ and *bla*_*OXA*_. Primer sequences and reaction conditions are provided in Supplementary Material 1. The PCR reaction system contained 1 μL of DNA template, 12.5 μL of Taq enzyme, 1 μL each of upstream and downstream primers, and 9.5 μL of ddHH_2_O, yielding a total reaction volume of 25 μL. The reaction conditions were as follows: initial denaturation at 94 °C for 5 min; denaturation at 94 °C for 45 s, annealing at 55°C for 45 s, and extension at 72 °C for 60 s for 30 cycles; and final extension at 72°C for 10 min. A 5 μL aliquot of the PCR product was subjected to electrophoresis on a 1.5% agarose gel (110 V, 20 min).

### Statistical analysis

Data management and statistical analyses were performed using Microsoft Excel and IBM SPSS Statistics 27.0. Categorical variables were presented as counts and percentages. Between-group comparisons were conducted using Pearson’s chi-square test or Fisher’s exact test, with the latter applied when expected cell counts were < 5. All tests were two-tailed.

Odds ratios (ORs) and 95% confidence intervals (CIs) were calculated to assess the associations between grouping and study outcomes. The Haldane–Anscombe correction (0.5 added to all cells) was used for analyses involving zero cells to stabilize OR and 95% CI estimation. Comparisons with consistent outcomes in both groups were excluded from statistical testing, and ORs were not calculated for universal antimicrobial resistance in both groups.

For antimicrobial susceptibility testing, intermediate results for each antibiotic were not classified as resistant. The Bonferroni correction was used to adjust for multiple comparisons in each analytical set. Statistical significance was defined as a corrected *P* value < 0.05.

## Results

### Distribution of clinical specimen types and departmental sources

**FIGURE 1:**
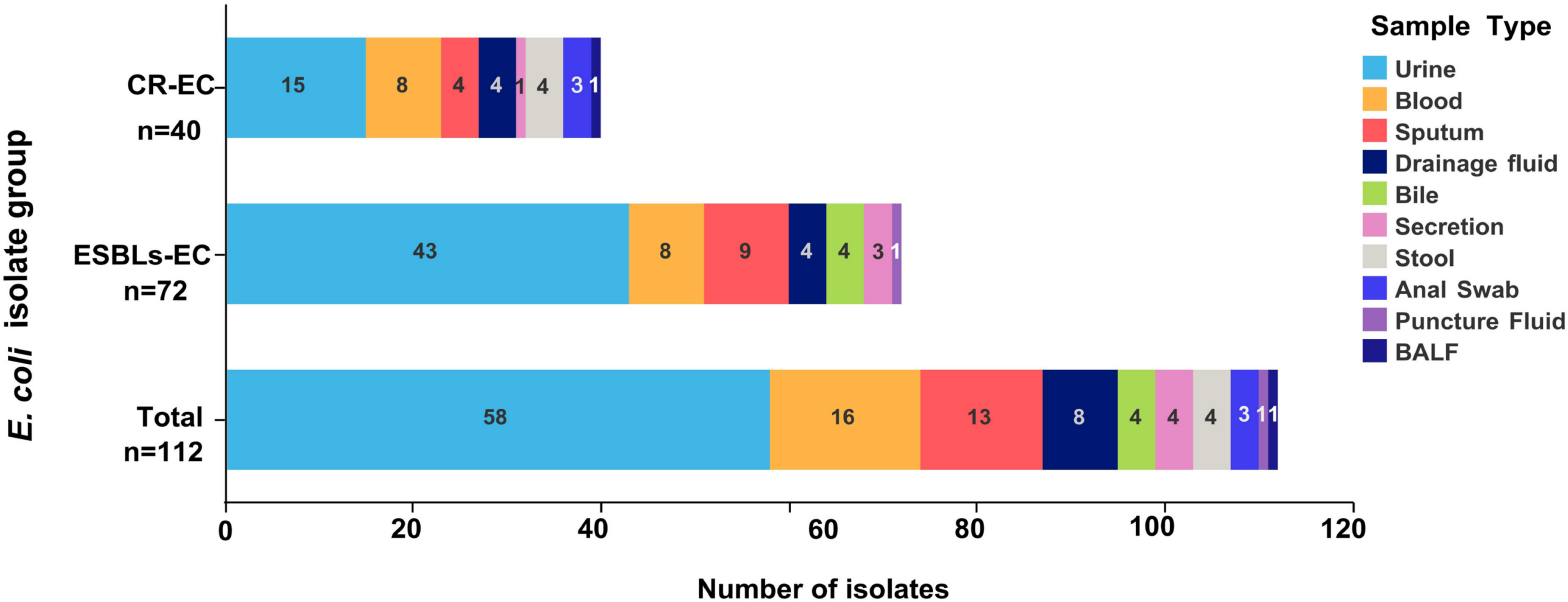
Distribution of clinical specimen types among ESBLs-EC and CR-EC isolates. The stacked bar chart illustrates the distribution of isolates by specimen type across the overall strain collection (*n* = 112), the ESBL-EC subgroup (*n* = 72), and the CR-EC subgroup (*n* = 40). Numerals inside the bars denote isolate counts. BALF, bronchoalveolar lavage fluid.

**FIGURE 2:**
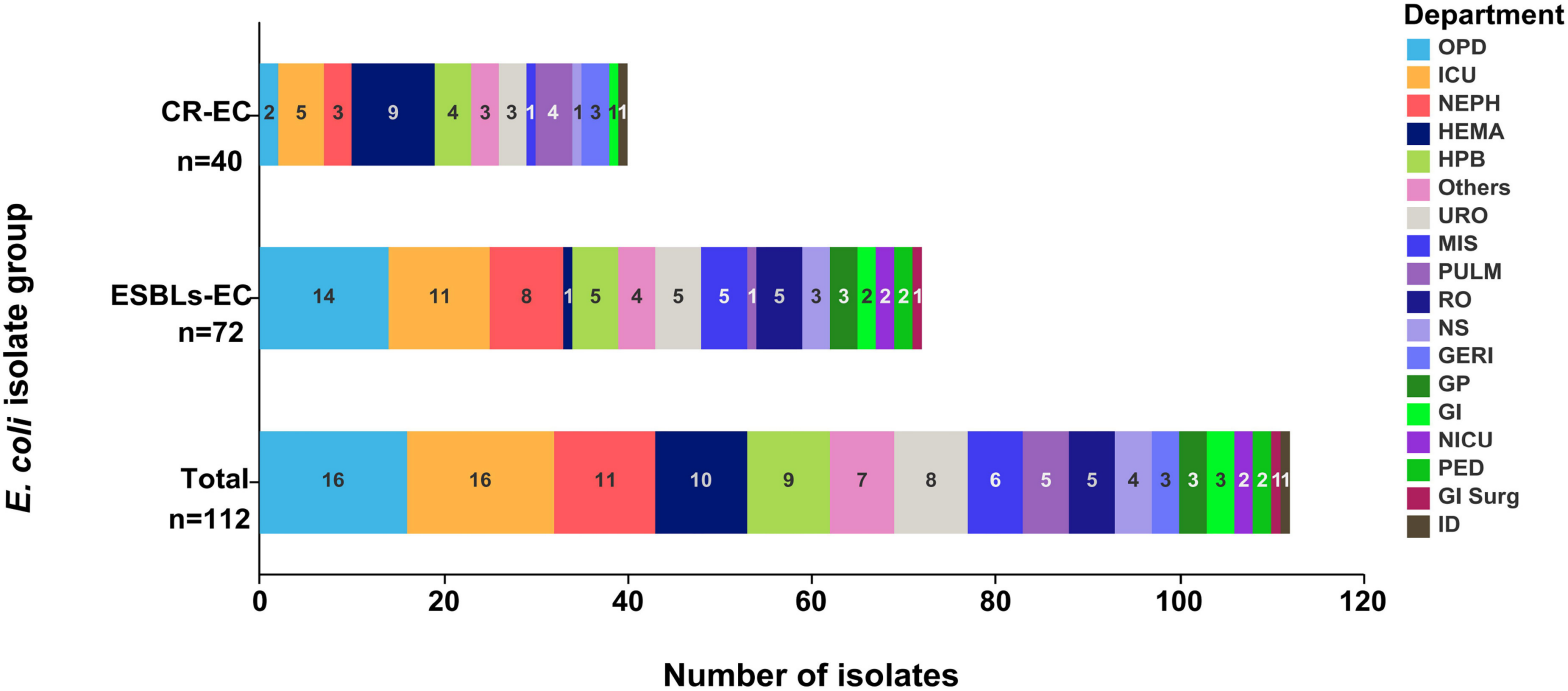
Distribution of ESBLs-EC and CE-EC isolates by department of origin. The stacked bar chart illustrates the distribution of isolates across hospital departments in the total collection (*n* = 112), the ESBL-EC subgroup, and the CR-EC subgroup (*n* = 40). Numbers within the bars represent isolate counts. ICU, intensive care unit; HEMA, hematology; MIS, minimally invasive surgery; HPB, hepatobiliary surgery; GI Surg, gastrointestinal surgery; OPD, outpatient department; NICU, neonatal intensive care unit; URO, urology; NS, neurosurgery; PULM, respiratory medicine; NEPH, nephrology; GI, gastroenterology; PED, pediatrics; RO, radiation oncology; GP, general practice; ORTH, orthopedics; ENDO, endocrinology; GERI, geriatrics; ID, infectious diseases; OB, obstetrics; RHEUM, rheumatology; CARD, cardiology; GYN, gynecology. In the total group, “Others” included seven departments with one isolate each: Thoracic Surgery, Orthopedics, Obstetrics, Rheumatology, Cardiology, Gynecology, and Endocrinology. In the ESBLs-EC group, “Others” included four departments with one isolate each: Orthopedics, Endocrinology, Gynecology, and Thoracic Surgery. In the CR-EC group, “Others” included three departments with one isolate each: Rheumatology, Obstetrics, and Cardiology.

Among the isolated E. coli strains, urine was the predominant specimen source, with 58 isolates (51.79%), followed by blood with 16 isolates (14.29%), sputum with 13 isolates (11.61%), drainage fluid with 8 isolates (7.14%), and bile with 4 isolates (3.57%) (Figure 1). Departmental distribution showed that the largest numbers of isolates were obtained from the outpatient department and intensive care unit (ICU), with 16 cases each (14.29% for both), followed by nephrology with 11 cases (9.82%), hematology with 10 cases (8.93%), and hepatobiliary surgery with 9 cases (8.04%) (Figure 2). Urine ranked first among specimen types in both groups, including 43 ESBLs-EC isolates (59.72%) and 15 CR-EC isolates (37.50%) (Figure 1). By department, ESBLs-EC isolates were mainly recovered from the outpatient department (14 cases, 19.44%), whereas CR-EC isolates were mainly recovered from hematology (9 cases, 22.50%) (Figure 2).

### Detection proportions of eSBLs-eC and CR-EC in outpatient and inpatient samples

Among outpatient samples, ESBLs-EC represented 87.50% (14/16) of isolates, while CR-EC represented 12.50% (2/16). In inpatient samples, ESBLs-EC accounted for 60.42% (58/96), and CR-EC accounted for 39.58% (38/96). The distribution of isolate categories differed significantly between outpatient and inpatient samples (χ^2^ = 4.38, *P* = 0.048). CR-EC isolates were more likely than ESBLs-EC isolates to be recovered from inpatient samples, although the 95% confidence interval was wide (OR = 4.59, 95% CI: 0.99–21.33) (Table 1).

**TABLE 1:** Detection rates of ESBLs-EC and CR-EC isolates in outpatient and inpatient samples.

Sources	Outpatient Sources	Inpatient Sources	*χ ^2^*	*P*	OR (95% CI)
	*n* (%) (*N* = 16)	*n* (%) (*N* = 96)			
ESBLs-EC	14 (87.5)	58 (60.42)	4.38	0.036	Reference
CR-EC	2 (12.5)	38 (39.58)			4.59 (0.99–21.33)

Data are shown as *n* (%). Percentages were calculated within each clinical setting column. *P-*values were calculated using Pearson’s chi-square test as appropriate. ORs represent the odds of inpatient origin in CR-EC isolates compared with ESBLs-EC isolates, with ESBLs-EC as the reference group.

### MLST typing

**FIGURE 3:**
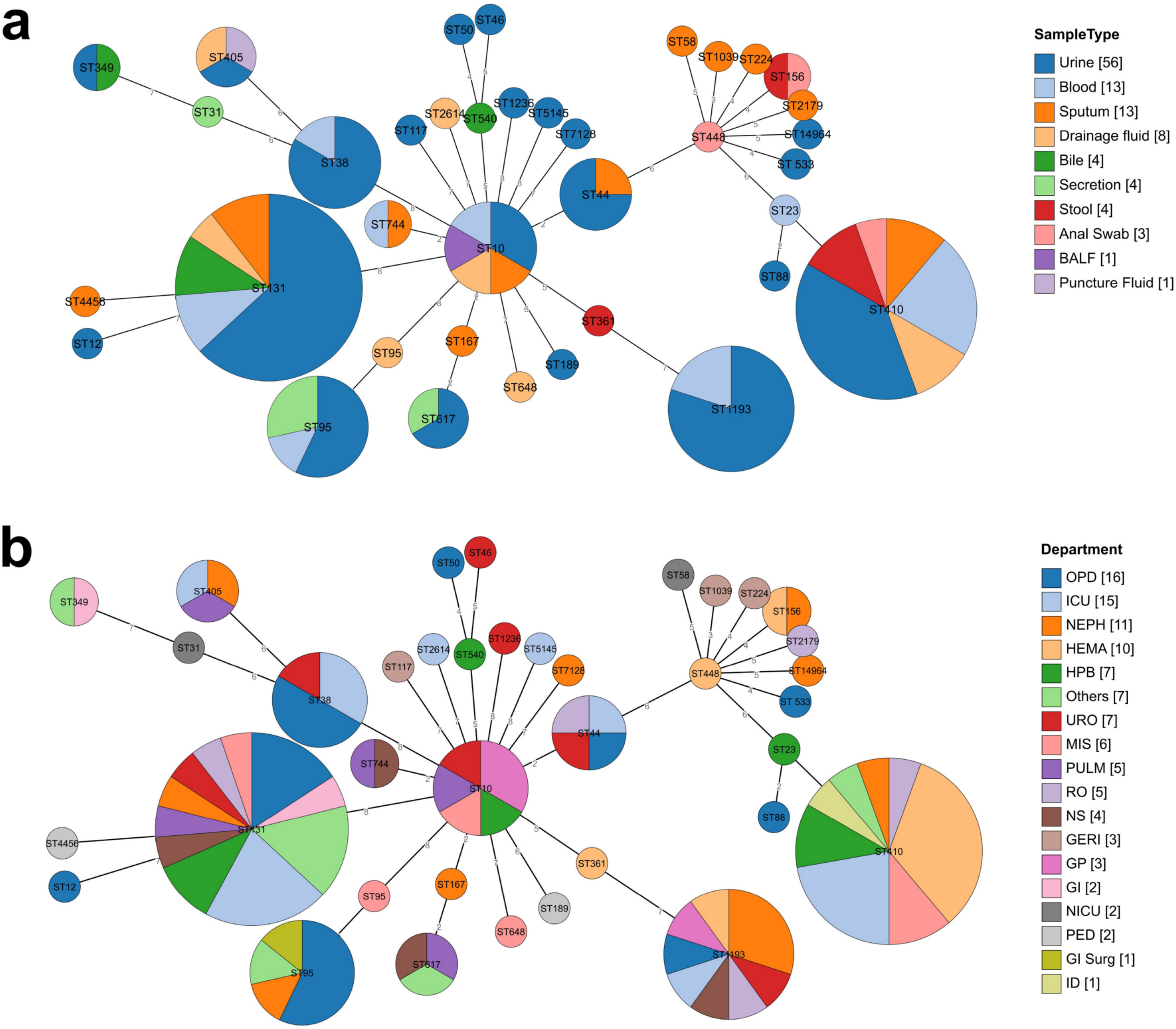
MLST-based GrapeTree analysis of clonal relationships among 112 *Escherichia coli* isolates. The GrapeTree network was constructed based on the allelic profiles of seven MLST housekeeping genes. Each node represents a sequence type, and node size indicates the number of isolates assigned to that ST. Branch labels indicate the number of allelic differences between connected STs. Nodes are colored according to sample type **(a)** and clinical department **(b)**. The network shows that the isolates were distributed across multiple clonal backgrounds, with ST131 and ST410 representing the major sequence types. In this figure, departments grouped under “Others” accounted for 7 isolates in total, with 1 isolate each from thoracic surgery, orthopedics, obstetrics, rheumatology, cardiology, gynecology, and endocrinology.

Seven-locus MLST data were analyzed in GrapeTree to reconstruct the clonal relationships among the 112 clinical *E. coli* isolates, with specimen type and clinical department used for annotation (Figure 3). Sequence type (ST) assignment was successful for 107 isolates, whereas 5 isolates did not yield valid typing results, including 1 ESBLs-EC isolate and 4 CR-EC isolates. Among ESBLs-EC isolates, 25 STs were identified. ST131 was the most frequent type (18/71, 25.35%), followed by ST1193 (9/71, 12.68%). Among CR-EC isolates, 20 STs were identified. ST410 was the most frequent type (12/36, 33.33%), followed by ST617 (3/36, 8.33%). The GrapeTree network revealed marked clonal heterogeneity, with isolates dispersed across multiple ST clusters rather than concentrated within a single dominant lineage. ST131 and ST410 formed the 2 largest nodes in the network, corresponding to their predominance in the ESBLs-EC and CR-EC groups, respectively. Their separation into different network regions indicates that these 2 resistant populations arose from distinct clonal backgrounds. Other STs, including ST95, ST1193, ST38, and ST10-related variants, were also detected, supporting the coexistence of multiple evolutionary lineages in the local *E. coli* population. Dominant STs were recovered from diverse specimen types and multiple departments, indicating that the *E. coli* population in this hospital was shaped by both leading clonal backgrounds and broad lineage diversity, rather than by a single local clonal expansion event.

### Detection of virulence genes

The detection rates of virulence genes were compared between ESBLs-EC and CR-EC isolates. After Bonferroni correction, the iron acquisition system genes *fyuA* and *iutA* retained statistically significant between-group differences, with higher positivity in ESBLs-EC isolates than in CR-EC isolates (72.22% vs. 40.00%, corrected *P* = 0.018; 69.44% vs. 37.50%, corrected *P* = 0.018). The pathogenicity island-associated gene PAI was also more prevalent in ESBLs-EC isolates (40.28% vs. 0.00%, corrected *P* < 0.001).

**FIGURE 4:**
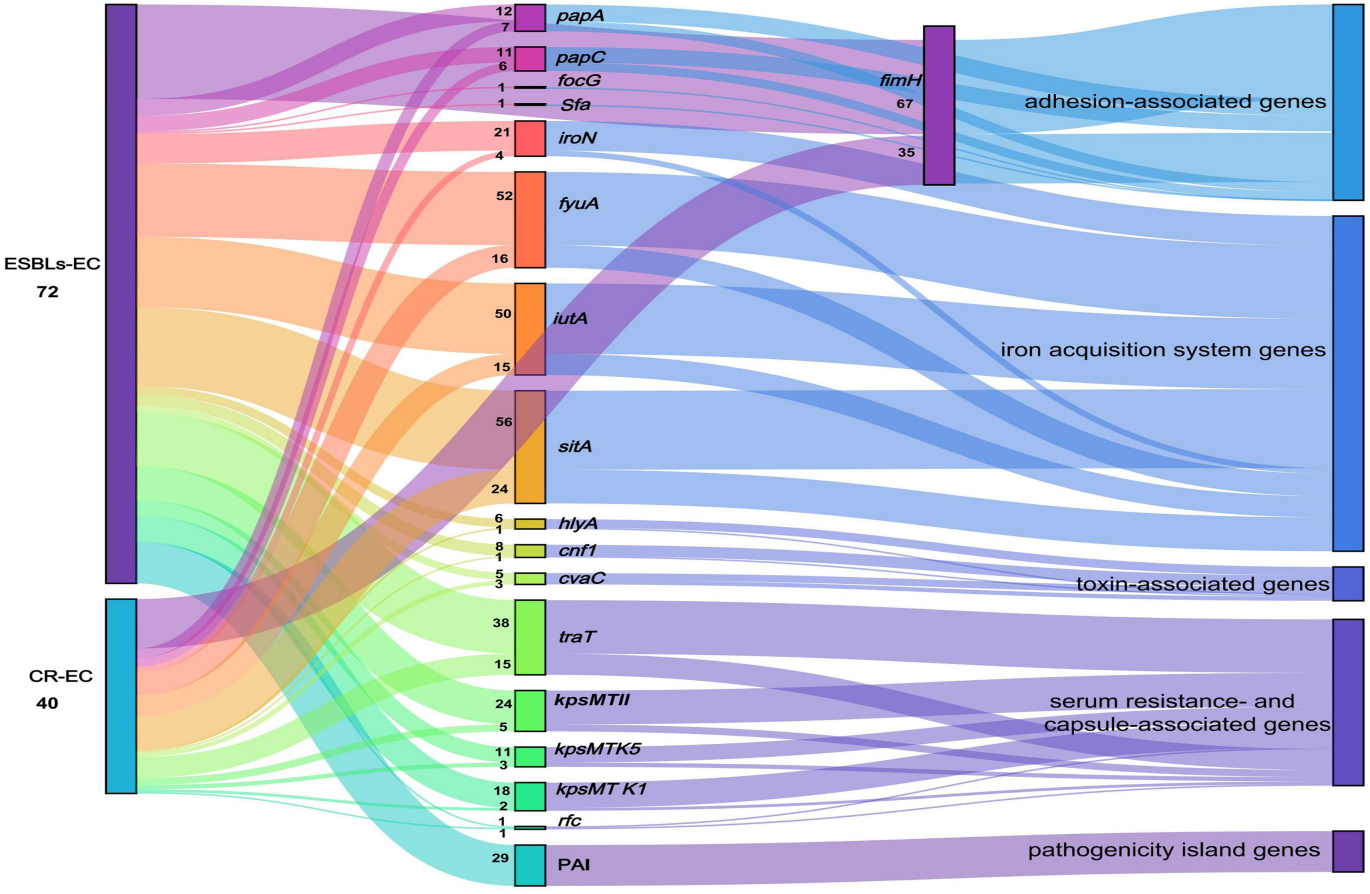
Detection rates of virulence genes in 112 *Escherichia coli* isolates. Sankey diagram illustrating the distribution of virulence-associated genes among ESBLs-EC (*n* = 72) and CR-EC (*n* = 40). The left column represents the two isolate groups, the middle column shows individual virulence genes, and the right column indicates their functional categories, including adhesion-associated genes, iron acquisition system genes, toxin-associated genes, serum resistance and capsule-associated genes, and pathogenicity island genes (PAI). The width of each flow is proportional to the number of isolates carrying the corresponding virulence determinant.

Although the unadjusted P values for iroN and kpsMTK1 suggested between-group differences, these associations were no longer significant after correction for multiple comparisons, with corrected P values of 0.558 and 0.162, respectively. For the remaining adhesin, toxin, and immune evasion genes, no significant differences in detection proportions were observed between the 2 groups either before or after correction, with all corrected P values equal to 1.000 (Figure 4 and Table 2).

**TABLE 2:** Distribution and detection rates of major virulence genes in ESBLs-EC and CR-EC isolates.

Virulence genes		ESBLs-EC	CR-EC	χ ^2^	*P*	OR (95% CI)	*P*_corrected
		*n* (%) (*N* = 72)	*n* (%) (*N* = 40)				
Adhesion-associated genes	*fimH*	67 (93.1)	35 (87.5)	–	0.326^†^	1.9 (0.41–8.88)	1.000
	*papA*	12 (16.7)	7 (17.5)	0.01	0.910	0.94 (0.34–2.63)	1.000
	*papC*	11 (15.3)	6 (15.0)	0.00	0.969	1.02 (0.35–3.01)	1.000
	*focG*	1 (1.4)	0 (0.0)	–	1.000^†^	0.55 (0.01–14.00)	1.000
	*sfa*	1 (1.4)	0 (0.0)	–	1.000^†^	0.55 (0.01–14.00)	1.000
Iron acquisition system genes	*iroN*	21 (29.2)	4 (10.0)	5.45	0.020	3.71 (1.17–11.72)	0.353
	*fyuA*	52 (72.2)	16 (40.0)	11.19	< 0.001	3.9 (1.72–8.82)	0.015
	*iutA*	50 (69.4)	15 (37.5)	10.77	0.001	3.79 (1.68–8.54)	0.019
	*sitA*	56 (77.8)	24 (60.0)	3.98	0.046	2.33 (1.01–5.42)	0.828
Toxin-associated genes	*hly*	6 (8.3)	1 (2.5)	–	0.418^†^	3.51 (0.4–167.07)	1.000
	*cnfL*	8 (11.1)	1 (2.5)	–	0.154^†^	4.82 (0.61–221.55)	1.000
	*cvaC*	5 (6.9)	3 (7.5)	–	1.000^†^	0.92 (0.17–6.26)	1.000
Serum resistance- and capsule-associated genes	*traT*	38 (52.8)	15 (37.5)	2.41	0.121	1.86 (0.85–4.1)	1.000
	*kpsMTII*	24 (33.3)	5 (12.5)	5.82	0.016	3.5 (1.22–10.08)	0.286
	*kpsK5*	11 (15.3)	3 (7.5)	1.42	0.233	2.22 (0.58–8.5)	1.000
	*kpsK1*	18 (25.0)	2 (5.0)	7.01	0.008	6.33 (1.39–28.92)	0.146
	*rfc*	1 (1.4)	1 (2.5)	–	1.000^†^	0.55 (0.01–44.21)	1.000
the pathogenicity island gene	PAI	29 (40.3)	0 (0.0)	21.74	< 0.001	54.93 (3.25–928.81)	< 0.001

Note: Data are shown as *n* (%). *P*-values were calculated using Pearson’s χ^2^ test or Fisher’s exact test, as appropriate. Fisher’s exact test was used when expected cell counts were less than 5. χ^2^ values are reported only for comparisons assessed using Pearson’s chi-square test.

† Fisher’s exact test. ORs represent the odds of gene positivity in CR-EC isolates compared with ESBLs-EC isolates, with ESBLs-EC as the reference group. For comparisons with zero observed cells, ORs and 95% CIs were calculated using the Haldane–Anscombe correction by adding 0.5 to each cell. Corrected *P* values were obtained via the Bonferroni method for 18 valid comparisons. Statistical significance was defined as *P* < 0.05.

### Detection of resistance genes

Resistance gene testing showed that *bla*_*CTX–M*_ was the predominant resistance gene among ESBLs-EC isolates, with a significantly higher detection proportion than that in CR-EC isolates (93.1% [67/72] vs. 35.0% [14/40], *P* < 0.001, corrected *P* < 0.001). In contrast, *bla*_*NDM*_ was detected only in CR-EC isolates (62.5% [25/40] vs. 0.0% [0/72], *P* < 0.001, corrected *P* < 0.001), indicating a strong association between *bla*_*NDM*_ carriage and the CR-EC phenotype. *bla*_*KPC*_ was likewise found only in CR-EC isolates, although at a low frequency (7.5% [3/40]), and the difference did not remain statistically significant after Bonferroni correction (corrected *P* = 0.172). For *bla*_*TEM*_, no significant difference was identified between the 2 groups (52.5% [21/40] vs. 41.7% [30/72], *P* = 0.270). In addition, *bla*_*SHV*_, *bla*_*IMP*_, *bla*_*VIM*_ and *bla*_*OXA*_ were not detected in either group (Table 3).

**TABLE 3:** Detection rates of resistance genes in ESBLs-EC and CR-EC isolates.

Resistance Gene	ESBLs-EC (Ref)	CR-EC	*χ 2*	*P*	OR (95%CI)	*P*_corrected
	*n* (%) (*N* = 72)	*n* (%) (*N* = 40)				
*bla*_TEM_	30 (41.70)	21 (52.50)	1.22	0.270	1.55 (0.71–3.37)	1.000
*bla*_SHV_	0 (0.0)	0 (0.0)	–	–	–	–
*bla*_CTX–M_	67 (93.10)	14 (35.00)	43.3	<0.001	0.04 (0.01–0.12)	<0.001
*bla*_NDM_	0 (0.0)	25 (62.50)	57.9	<0.001	238.55 (13.77–4132.79)	<0.001
*bla*_KPC_	0 (0.0)	3 (7.50)	–	0.043^†^	13.53 (0.68–268.95)	0.172
*bla*_IMP_	0 (0.0)	0 (0.0)	–	–	–	–
*bla*_VIM_	0 (0.0)	0 (0.0)	–	–	–	–
*bla*_OXA_	0 (0.0)	0 (0.0)	–	–	–	–

Data are shown as *n* (%). *P*-values were calculated using Pearson’s chi-square test or Fisher’s exact test, as appropriate. Fisher’s exact test was used when expected cell counts were less than 5. χ^2^ values are reported only for comparisons assessed using Pearson’s chi-square test.

† Fisher’s exact test. ORs represent the odds of gene positivity in CR-EC isolates compared with ESBLs-EC isolates. For comparisons with zero observed cells, ORs and 95% CIs were calculated using the Haldane–Anscombe correction by adding 0.5 to each cell. Corrected *P*-values were calculated using the Bonferroni method for four valid comparisons. Genes with constant negative results in both groups were excluded from statistical analyses and multiple-comparison correction.

### Antimicrobial susceptibility testing

Intermediate susceptibility results were included in the total denominator and were not classified as resistant. Under this statistical rule, CR-EC isolates still showed significantly higher resistance proportions to most antimicrobial agents than ESBLs-EC isolates. All CR-EC isolates were resistant to cefotetan, cefepime, ceftazidime, ampicillin, ampicillin/sulbactam, ertapenem, imipenem, and piperacillin/tazobactam. After Bonferroni correction for multiple comparisons, resistance proportions remained significantly different between the 2 groups for cefotetan, cefepime, ceftazidime, ampicillin/sulbactam, ciprofloxacin, ertapenem, imipenem, piperacillin/tazobactam, amikacin, and tobramycin, with CR-EC isolates showing a higher risk of resistance to these agents.

Resistance to carbapenems was confined to CR-EC isolates. The resistance proportions to ertapenem and imipenem were both 100.0% in CR-EC isolates, whereas no resistance to either agent was observed among ESBLs-EC isolates (corrected *P* < 0.001). Resistance to ciprofloxacin was also markedly higher in CR-EC isolates than in ESBLs-EC isolates, at 97.5% (39/40) and 54.2% (39/72), respectively (corrected *P* < 0.001). CR-EC isolates also had higher resistance proportions to tobramycin (40.0% [16/40] vs. 9.7% [7/72], corrected *P* = 0.003) and amikacin (17.5% [7/40] vs. 0.0% [0/72], corrected *P* = 0.009). Although resistance to trimethoprim/sulfamethoxazole, nitrofurantoin, gentamicin, and levofloxacin was numerically higher in CR-EC isolates, the between-group differences were not statistically significant after Bonferroni correction. No significant difference was observed between the 2 groups for resistance to aztreonam, ceftriaxone, or cefazolin; by contrast, ampicillin resistance was universal in both groups (Table 4).

**TABLE 4:** Comparative analysis of antimicrobial resistance rates between ESBLs-EC and CR-EC isolates.

Antibiotic	Resistance breakpoint (mg/L)			ESBLs-EC	CR-EC	*χ ^2^*	*P*	OR (95%CI)	*P*_corrected
	S	I	R	*n* (%) (*N* = 72)	*n* (%) (*N* = 40)				
ATM	≤ 4	8	≥ 16	47 (65.3)	22 (55.0)	1.15	0.284	0.65 (0.30–1.43)	1.000
CTT	≤ 16	32	≥ 64	3 (4.2)	40 (100.0)	99.85	< 0.001	1608.43 (81.00–31900)	< 0.001
CRO	≤ 1	2	≥ 4	69 (95.8)	40 (100.0)	–	0.551^†^	4.08 (0.21–80.96)	1.000
FEP	≤ 2	–	≥ 16	15 (20.8)	40 (100.0)	–	< 0.001	300.48 (17.47–5166)	< 0.001
CAZ	≤ 4	8	≥ 16	29 (40.3)	40 (100.0)	38.78	< 0.001	119.44 (7.06–2019)	< 0.001
CFZ	≤ 2	4	≥ 8	70/72 (97.2)	40 (100.0)	–	0.537^†^	2.87 (0.13–61.31)	1.000
AMP	≤ 8	16	≥ 32	72/72 (100.0)	40 (100.0)	–	–	–	–
SAM	≤ 8/4	8/16	≥ 32/16	45 (62.5)	40 (100.0)	19.77	< 0.001	48.96 (2.89–828.2)	< 0.001
CIP	≤ 0.25	0.5	≥ 1	39 (54.2)	39 (97.5)	22.84	< 0.001	33.00 (4.30–253.9)	< 0.001
LEV	≤ 0.5	1	≥ 2	47 (65.3)	32 (80.0)	2.68	0.102	2.13 (0.85–5.30	1.000
ETP	≤ 0.5	1	≥ 2	0 (0.0)	40 (100.0)	112.00	< 0.001	11745.00 (228.7–603000)	< 0.001
IPM	≤ 1	2	≥ 4	0 (0.0)	40 (100.0)	112.00	< 0.001	11745.00 (228.7–603000)	< 0.001
TZP	≤ 8/4	–	≥ 32/4	2 (2.8)	40 (100.0)	103.70	< 0.001	2284.20 (107.0–48800)	< 0.001
SXT	≤ 2/38	–	≥ 4/76	40 (55.6)	32 (80.0)	6.69	0.010	3.20 (1.30–7.90)	0.180
NIT	≤ 32	64	≥ 128	1 (1.4)	6 (15.4)	–	0.008^†^	12.53 (1.45–108.31)	0.144
AMK	≤ 4	8	≥ 16	0 (0.0)	7 (17.5)	–	< 0.001^†^	32.46 (1.80–584.6)	0.009
GEN	≤ 2	4	≥ 8	27 (37.5)	25 (62.5)	6.46	0.011	2.78 (1.25–6.17)	0.198
TOB	≤ 2	4	≥ 8	7 (9.7)	16 (40.0)	14.45	< 0.001	6.19 (2.27–16.89)	0.003

Data are shown as *n* (%). Intermediate susceptibility results were included in the denominator but were not counted as resistant. *P*-values were calculated using Pearson’s chi-square test or Fisher’s exact test, as appropriate.

† Fisher’s exact test. χ^2^ values are reported only for comparisons assessed using Pearson’s chi-square test. ORs represent the odds of resistance in CR-EC isolates compared with ESBLs-EC isolates. For comparisons with zero observed cells, ORs and 95% CIs were calculated using the Haldane–Anscombe correction by adding 0.5 to each cell. Corrected values were calculated using the Bonferroni method for 18 antibiotics. AMP showed constant resistance in both groups; therefore, the OR was not estimable. ATM, aztreonam; CTT, cefotetan; CRO, ceftriaxone; FEP, cefepime; CFZ, cefazolin; CAZ, ceftazidime; AMP, ampicillin; SAM, ampicillin/sulbactam; CIP, ciprofloxacin; LEV, levofloxacin; ETP, ertapenem; IPM, imipenem; TZP, piperacillin/tazobactam; SXT, trimethoprim/sulfamethoxazole; NIT, nitrofurantoin; AMK, amikacin; GEN, gentamicin; TOB, tobramycin.

## Discussion

In this study, ESBLs-EC was detected more frequently in outpatient samples than in inpatient samples, a distribution consistent with the broad dissemination of ESBLs-EC across health care and community settings worldwide (Nguyen et al., 2024). By contrast, CR-EC was significantly more common in inpatient samples than in outpatient samples. From the perspective of sample distribution, the ICU yielded a higher recovery proportion than other departments, and inpatient-derived CR-EC isolates were mainly concentrated in hematology and the ICU. This pattern may be related to impaired host immunity in these patients (Wang et al., 2025). In ICU patients, invasive procedures such as mechanical ventilation and urinary catheterization are also common and may increase the risk of mucosal barrier disruption, creating opportunities for opportunistic pathogens to cause infection (Razazi et al., 2024). In addition, antimicrobial selective pressure may alter the intestinal microbiota and support dominant expansion of *E. coli* (Boscolo et al., 2022). Therefore, supportive care for immunocompromised patients should be optimized; for example, the application of granulocyte colony-stimulating factor (G-CSF) may reduce infection risk (Walti et al., 2023). Surveillance of drug-resistant bacteria should also be strengthened in high-risk wards such as the ICU, with priority given to noninvasive ventilation when clinically appropriate (Razazi et al., 2024), reduction of invasive procedures (Perez-Galera et al., 2023), and close prevention of in-hospital outbreaks of ESBLs-EC and CR-EC.

To characterize the clonal relationships among the 112 E. coli isolates, a 7-locus MLST-based allelic profile network was constructed with GrapeTree and annotated according to specimen type and clinical department (Figure 3). Among the 112 *E. coli* isolates subjected to ST analysis, 107 were successfully assigned to defined STs, whereas 5 isolates remained untypeable. In the allelic profile network, ST131 and ST410 formed the 2 largest nodes. These 2 high-frequency ST groups were located on different allelic branches in GrapeTree, indicating that they originated from different ancestral clones and that acquisition of resistance phenotypes likely reflected independent evolutionary events. Among ESBLs-EC isolates, ST131 was the predominant clone, accounting for 25.35%, which is consistent with the major ST profile of ESBLs-EC reported in many clinical studies in China and other countries (Li et al., 2021; Phan et al., 2022; Aydin et al., 2025). As a globally dominant clone of extraintestinal pathogenic *E. coli* (ExPEC), ST131 expanded rapidly after 2008 and reached a peak around 2020. It is closely associated with human infections and carries ESBL genes such as bla_CTX–M_ more frequently than other STs (Rana et al., 2026).

Among CR-EC isolates, ST410 was dominant, with a markedly higher proportion than other STs. ST410 is an emerging MDR clone. Previous data from hospitals in China during 2017–2021 showed that ST410 had become the most frequent ST among CR-EC isolates, with identification of the highly virulent B5/H24RxC clone, which had caused 2 independent outbreaks in children’s hospitals (Yao et al., 2025). In addition to ST131 and ST410, several minor STs were detected, including ST95, ST1193, ST38, and ST10-related variants. This finding is consistent with the study by Puljko et al. (2024) and supports the coexistence of multiple lineages within the local *E. coli* population.

Annotation by specimen type showed that the major STs were recovered from diverse clinical specimens, including urine, blood, sputum, and drainage fluid. Department-based annotation further indicated that dominant ST groups were distributed across internal medicine, surgery, intensive care medicine, and other departments, rather than being confined to a single ward. These findings suggest that the *E. coli* population in this hospital was shaped by both dominant clonal backgrounds and broader lineage diversity. It did not appear to result from a single local clonal expansion event, instead showing a complex structure characterized by “dominant clones plus diverse lineages,” which provides important evidence for understanding the evolutionary pathways of MDR bacteria and designing prevention and control strategies.

This study also identified several features in the virulence gene distribution of ESBLs-EC and CR-EC. The adhesin gene *fimH* showed the highest carriage proportion, followed by iron acquisition system genes, including *fyuA*, *sitA*, and *iutA*. This distribution pattern is consistent with the findings of Suarez-Yana et al. (2024), suggesting that type I fimbriae constitute a shared colonization basis in *E. coli*. These fimbriae mainly mediate bacterial adhesion and represent a key pathogenic factor after colonization during *E. coli* infection (Lopatto et al., 2024). The difference in the iron metabolism-related virulence gene *fyuA* indicates that ESBLs-EC may rely more strongly on the yersiniabactin system for iron acquisition, which may be related to its higher virulence profile. *sitA* belongs to the *SitABCD* iron acquisition system and is closely associated with extraintestinal invasive infections; its high carriage proportion may confer stronger pathogenic capacity in ESBLs-EC (Lara-Gutierrez et al., 2026). Serum resistance- and capsule-associated virulence genes (Chen et al., 2025), such as *traT*, *kpsMTII*, *kpsMTK1*, and *kpsMTK5*, were carried more frequently by ESBLs-EC than by CR-EC, with a statistically significant difference between the 2 groups. These genes mainly encode outer membrane proteins and capsular components, which may protect *E. coli* from host immune recognition after invasion of host cells and suppress host inflammatory responses (Souza et al., 2025). These findings suggest that ESBLs-EC may be more pathogenic than CR-EC (De Francesco et al., 2023; Paris et al., 2024). The research group will further validate the virulence difference between the 2 groups in mouse models.

PAI, the pathogenicity island, was completely absent in the CR-EC group, but reached 40.28% in the ESBLs-EC group, indicating that ESBLs-EC isolates were more likely to carry PIA. This finding suggests that CR-EC isolates may have lost virulence factors during evolution, resulting in a “high-resistance, low-virulence” phenotype. This study did not show that carbapenem resistance in CR-EC was accompanied by broad loss of virulence genes. However, the complete absence of PAI and the low carriage of the *kps* gene cluster suggest that CR-EC may maintain survival through a “resistance compensation” strategy rather than relying on traditional virulence factors.

Regarding resistance genes, ESBLs-EC isolates were mainly characterized by *bla*_CTX–M_, consistent with the findings reported by Tian et al., 2020. ESBL-related resistance genes are shifting from the *bla*_SHV_ and *bla*_TEM_ genotypes toward the *bla*_CTX–M_ genotype, which may be associated with the ISEcp transfer element upstream of *bla*_CTX–M_ (Palzkill, 2018). Previous studies have demonstrated that the distinctive ISEcp1-*bla*_*CTX–M*_-orf477 genetic structure specific to *bla*_CTX–M_ significantly increases its horizontal transfer efficiency (Sultan et al., 2022). By contrast, CR-EC isolates were mainly represented by *bla*_NDM_, a pattern consistent with the results of Shafiq et al. (2024).

Notably, approximately 30% of CR-EC isolates in the present study did not harbor any of the screened carbapenemase genes, despite exhibiting phenotypic resistance to carbapenems. This finding suggests that carbapenem resistance in these isolates may be mediated by mechanisms other than the commonly detected carbapenemases. Previous studies have demonstrated that reduced outer membrane permeability caused by the loss or downregulation of porins such as *OmpC* and *OmpF* can significantly decrease carbapenem uptake (Rodriguez-Villodres et al., 2024; Wang et al., 2024). In addition, overexpression of multidrug efflux pumps may contribute to reduced intracellular antibiotic accumulation. Hyperproduction of AmpC β-lactamases or ESBLs, particularly when combined with porin deficiency, has also been reported as an important non-carbapenemase-mediated mechanism of carbapenem resistance in *Escherichia coli* (Wei et al., 2021). Furthermore, constrained by inherent limitations of PCR detection, this study only screened prevalent, well-documented carbapenemase genes and could not fully characterize other resistance-associated molecular signatures. To mitigate this research limitation, our group will integrate whole-genome sequencing with bioinformatic analyses once sufficient funding is obtained, to systematically dissect various non-enzymatic molecular mechanisms responsible for carbapenem resistance at the genomic level.

Antimicrobial susceptibility testing in this study showed that ESBLs-EC isolates carrying *bla*_CTX–M_ had resistance proportions above 90% to cephalosporins, including ceftriaxone and cefazolin, and to the penicillin ampicillin. This finding is directly related to the hydrolytic inactivation of β-lactam agents by ESBLs (Hinchliffe et al., 2022). These isolates, however, retained relatively high susceptibility to cefotetan, carbapenems including imipenem and ertapenem, amikacin, and nitrofurantoin, suggesting that these agents may serve as preferred therapeutic options for community-acquired ESBLs-EC infections in Ningbo.

CR-EC isolates carrying *bla*_NDM_ showed a pan-resistant profile. Except for relatively low resistance proportions to amikacin and nitrofurantoin, these isolates showed resistance proportions of 40%–65% to tobramycin, gentamicin, and aztreonam; resistance proportions as high as 80% to trimethoprim/sulfamethoxazole and levofloxacin; and resistance proportions above 97.5% to the remaining commonly prescribed clinical antimicrobial agents. These findings reflect an MDR phenotype mediated by carbapenem resistance genes. Between-group analysis further showed that CR-EC isolates had significantly higher resistance proportions than ESBLs-EC isolates to carbapenems, including ertapenem and imipenem; β-lactam/β-lactamase inhibitor combinations, including ampicillin/sulbactam; quinolones, including ciprofloxacin; and aminoglycosides, including tobramycin and amikacin. These differences were statistically significant and closely matched the resistance gene profiles of the 2 isolate groups, further indicating that resistance genotype is a key determinant of antimicrobial resistance phenotype.

## Conclusion

Taken together, antimicrobial resistance phenotyping, resistance gene typing, and virulence gene characterization of clinically isolated *E. coli* in Ningbo showed that the currently circulating *E. coli* population in this region is mainly composed of ESBLs-EC carrying *bla*_*CTX–M*_-type ESBLs and CR-EC carrying *bla*_*NDM*_-type carbapenem-resistant metallo-β-lactamases. This study identified a clear resistance–virulence trade-off. Under the dual influence of long-term antimicrobial selective pressure in clinical settings and bacterial adaptive evolution, resistance genes and virulence genes in *E. coli* appear to have diverged into 2 distinct and non-overlapping resistance–virulence evolutionary trajectories. First, ESBLs-EC represents a “lower-resistance, higher-virulence” evolutionary pattern. These isolates have a relatively narrow resistance spectrum and retain partial susceptibility to cephamycins, aminoglycosides, and carbapenems, yet they carry abundant virulence factors, such as adhesins, invasins, and hemolysins, and exhibit stronger host colonization and invasive capacity, which may favor their dissemination in community settings. Second, CR-EC represents a “high-resistance, low-virulence” evolutionary pattern. These isolates carry carbapenem resistance genes and show broad resistance to multiple classes of antimicrobial agents, including carbapenems, whereas their virulence gene carriage is markedly reduced and their virulence phenotype is relatively weak. Their transmission and spread may therefore depend more strongly on hospital-associated clinical procedures, such as invasive device placement and inappropriate antimicrobial exposure, often leading to clustered nosocomial infections or even outbreaks.

In clinical treatment, unnecessary carbapenem exposure should be avoided for community-acquired ESBLs-EC infections, and antimicrobial agents confirmed as susceptible by susceptibility testing should be prioritized. For hospital-acquired CR-EC infections, hierarchical antimicrobial stewardship should be strictly implemented, and nonessential carbapenem use should be restricted to reduce evolutionary selection pressure on resistant strains. At the community level, health education should be strengthened to reduce fecal–oral transmission of highly virulent ESBLs-EC among populations. At the hospital level, disinfection and sterilization of invasive medical devices should be reinforced, contact isolation measures should be implemented, and hand hygiene among health care workers should be standardized to block cross-transmission of CR-EC within hospital settings. All strains enrolled in this study were derived from a single center, which cannot fully reflect the overall molecular epidemiological characteristics and evolutionary patterns of *Escherichia coli* circulating in Ningbo. Multicenter and large-scale epidemiological screening will be conducted when conditions permit.

## Data Availability

The original contributions presented in this study are included in the article/Supplementary material, further inquiries can be directed to the corresponding authors.

## Footnotes

1
https://pubmlst.org/organisms/escherichia-spp

## References

[B1] Aydin E. Celebi S. Celebi O. Celebi D. Taghizadehghalehjoughi A. (2025). Investigations of *Escherichia coli* ST131 and H30Rx subclone from clinical samples. *Acta Microbiol. Immunol. Hung.* 72 226–236. 10.1556/030.2025.02710 41026537

[B2] Boscolo A. Sella N. Pettenuzzo T. De Cassai A. Crociani S. Schiavolin C. et al. (2022). Multidrug-resistant and extended-spectrum beta-lactamase gram-negative bacteria in bilateral lung transplant recipients: Incidence, risk factors, and in-hospital mortality. *Chest* 162 1255–1264. 10.1016/j.chest.2022.06.046 35850288

[B3] Chen N. Bukys A. Lundgren C. A. K. Deme J. C. El Sayyed H. Kapanidis A. N. et al. (2025). Structure of the conjugation surface exclusion protein TraT. *Commun. Biol.* 8:1702. 10.1038/s42003-025-09102-8 41299015 PMC12658005

[B4] Das B. J. Singha K. M. Wangkheimayum J. Chanda D. D. Bhattacharjee A. (2023). Emergence of carbapenem-resistant enterobacterales co-harboring blaOXA-78 and blaOXA-58 from India. *Ann. Clin. Microbiol. Antimicrob.* 22:79. 10.1186/s12941-023-00635-6 37679795 PMC10486080

[B5] De Francesco M. A. Bertelli A. Corbellini S. Scaltriti E. Risso F. Allegri R. et al. (2023). Emergence of pandemic clonal lineage sequence types 131 and 69 of extraintestinal *Escherichia coli* as a cause of meningitis: Is it time to revise molecular assays? *Microbiol. Spectr.* 11:e0327422. 10.1128/spectrum.03274-22 36786647 PMC10100906

[B6] Hinchliffe P. Tooke C. L. Bethel C. R. Wang B. Arthur C. Heesom K. J. et al. (2022). Penicillanic acid sulfones inactivate the extended-spectrum beta-lactamase CTX-M-15 through formation of a serine-lysine cross-link: An alternative mechanism of beta-lactamase inhibition. *mBio* 13:e0179321. 10.1128/mbio.01793-21 35612361 PMC9239225

[B7] Huang J. Lv C. Li M. Rahman T. Chang Y. F. Guo X. et al. (2024). Carbapenem-resistant *Escherichia coli* exhibit diverse spatiotemporal epidemiological characteristics across the globe. *Commun. Biol.* 7:51. 10.1038/s42003-023-05745-7 38184739 PMC10771496

[B8] Ilmavirta H. Ollgren J. Raisanen K. Kinnunen T. Hakanen A. J. Jalava J. et al. (2023). Increasing proportions of extended-spectrum beta-lactamase-producing isolates among *Escherichia coli* from urine and bloodstream infections: Results from a nationwide surveillance network, Finland, 2008 to 2019. *Euro Surveill.* 28:2200934. 10.2807/1560-7917.Es.2023.28.43.2200934 37883040 PMC10604539

[B9] Lara-Gutierrez J. Nguyen J. Mcilvin M. R. Sugiyama I. Landry Z. C. Alcolombri U. et al. (2026). Bacterial iron acquisition by *Escherichia coli* is facilitated by amino acid complexation in a rapid-renewal environment. *Proc. Natl. Acad. Sci. U. S. A.* 123:e2520431123. 10.1073/pnas.2520431123 41671182 PMC12912997

[B10] Li D. Wyrsch E. R. Elankumaran P. Dolejska M. Marenda M. S. Browning G. F. et al. (2021). Genomic comparisons of *Escherichia coli* ST131 from Australia. *Microb. Genom.* 7:000721. 10.1099/mgen.0.000721 34910614 PMC8767332

[B11] Li Y. Sun X. Dong N. Wang Z. Li R. (2024a). Global distribution and genomic characteristics of carbapenemase-producing *Escherichia coli* among humans, 2005-2023. *Drug Resist. Updat.* 72:101031. 10.1016/j.drup.2023.101031 38071860

[B12] Li Y. Zhang Y. Sun X. Wu Y. Yan Z. Ju X. et al. (2024b). National genomic epidemiology investigation revealed the spread of carbapenem-resistant *Escherichia coli* in healthy populations and the impact on public health. *Genome Med.* 16:57. 10.1186/s13073-024-01310-x 38627827 PMC11020349

[B13] Lopatto E. D. B. Santiago-Borges J. M. Sanick D. A. Malladi S. K. Azimzadeh P. N. Timm M. W. et al. (2024). Monoclonal antibodies targeting the FimH adhesin protect against uropathogenic *E. coli* UTI. *bioRxiv* [Preprint] 10.1101/2024.12.10.627638 40540557 PMC12180514

[B14] Macesic N. Uhlemann A. C. Peleg A. Y. (2025). Multidrug-resistant Gram-negative bacterial infections. *Lancet* 405 257–272. 10.1016/s0140-6736(24)02081-6 39826970

[B15] Musicha P. Beale M. A. Cocker D. Oruru F. A. Zuza A. Salifu C. et al. (2026). Transmission of extended spectrum beta-lactamase-producing *Escherichia coli* and antimicrobial resistance gene flow across one health compartments in eastern Africa: A whole-genome sequence analysis from a prospective cohort study. *Lancet Microbe* 7:101224. 10.1016/j.lanmic.2025.101224 41314227 PMC12888559

[B16] Nguyen M. N. Gladstone B. P. De Angelis G. Biggel M. Xavier B. B. Lammens C. et al. (2024). Tracing carriage, acquisition, and transmission of ESBL-producing *Escherichia coli* over two years in a tertiary care hospital. *Genome Med.* 16:151. 10.1186/s13073-024-01424-2 39707490 PMC11660618

[B17] Palzkill T. J. F. I. M. B. (2018). Structural and mechanistic basis for extended-spectrum drug-resistance mutations in altering the specificity of TEM, CTX-M, and KPC β-lactamases. *Front. Mol. Biosci.* 5:16. 10.3389/fmolb.2018.00016 29527530 PMC5829062

[B18] Paris T. Kiss A. Signor L. Lutfalla G. Blaise M. Boeri Erba E. et al. (2024). The IbeA protein from adherent invasive *Escherichia coli* is a flavoprotein sharing structural homology with FAD-dependent oxidoreductases. *FEBS J.* 291 177–203. 10.1111/febs.16969 37786987

[B19] Perez-Galera S. Bravo-Ferrer J. M. Paniagua M. Kostyanev T. De Kraker M. E. A. Feifel J. et al. (2023). Risk factors for infections caused by carbapenem-resistant Enterobacterales: An international matched case-control-control study (EURECA). *EClinicalMedicine* 57:101871. 10.1016/j.eclinm.2023.101871 36895801 PMC9989660

[B20] Phan M. D. Peters K. M. Alvarez Fraga L. Wallis S. C. Hancock S. J. Nhu N. T. K. et al. (2022). Plasmid-mediated ciprofloxacin resistance imparts a selective advantage on *Escherichia coli* ST131. *Antimicrob. Agents Chemother.* 66:e0214621. 10.1128/aac.02146-21 34780264 PMC8765324

[B21] Pierce V. M. Bhowmick T. Simner P. J. (2023). Guiding antimicrobial stewardship through thoughtful antimicrobial susceptibility testing and reporting strategies: An updated approach in 2023. *J. Clin. Microbiol.* 61:e0007422. 10.1128/jcm.00074-22 37768094 PMC10662363

[B22] Puljko A. Babic I. Rozman S. D. Barisic I. Jelic M. Maravic A. et al. (2024). Treated municipal wastewater as a source of high-risk and emerging multidrug-resistant clones of *E. coli* and other Enterobacterales producing extended-spectrum beta-lactamases. *Environ. Res.* 243:117792. 10.1016/j.envres.2023.117792 38048868

[B23] Quan J. Dai H. Liao W. Zhao D. Shi Q. Zhang L. et al. (2021). Etiology and prevalence of ESBLs in adult community-onset urinary tract infections in East China: A prospective multicenter study. *J. Infect.* 83 175–181. 10.1016/j.jinf.2021.06.004 34116075

[B24] Rana C. Vikas V. Narwal N. Korpela K. De S. (2026). Dynamics of extended spectrum cephalosporin and carbapenem resistance genes in *Escherichia coli*: An emerging environmental hazard. *J. Hazard. Mater.* 509:141955. 10.1016/j.jhazmat.2026.141955 41946245

[B25] Razazi K. Luyt C. E. Voiriot G. Rouze A. Garnier M. Ferre A. et al. (2024). Ventilator-associated pneumonia related to extended-spectrum beta-lactamase producing Enterobacterales during severe acute respiratory syndrome coronavirus 2 infection: Risk factors and prognosis. *Crit. Care* 28:131. 10.1186/s13054-024-04906-2 38641851 PMC11031867

[B26] Rios E. Del Carmen Lopez Diaz M. Culebras E. Rodriguez-Avial I. Rodriguez-Avial C. (2022). Resistance to fosfomycin is increasing and is significantly associated with extended-spectrum beta-lactamase-production in urinary isolates of *Escherichia coli*. *Med. Microbiol. Immunol.* 211 269–272. 10.1007/s00430-022-00749-2 36056943 PMC9618510

[B27] Rodriguez-Villodres A. Ortiz De La Rosa J. M. Galvez-Benitez L. Gascon M. L. Penalva G. Dorado Pardo F. J. et al. (2024). Survival of infection with TEM beta-lactamase-producing *Escherichia coli* with Pan-beta-lactam resistance. *J. Infect.* 89:106268. 10.1016/j.jinf.2024.106268 39278274

[B28] Shafiq M. Ahmed I. Saeed M. Malik A. Fatima S. Akhtar S. et al. (2024). Predominance of *bla*_*NDM*_- and *bla*_*IMP*_-harboring *Escherichia coli* belonging to clonal complexes 131 and 23 in a major university hospital. *Medicina* 60:1528. 10.3390/medicina60091528 39336569 PMC11434522

[B29] Souza S. S. R. Piper K. R. Ikhimiukor O. O. Marcovici M. M. Zac Soligno N. I. Harmon A. J. et al. (2025). Variants of beta-lactamase-encoding genes are disseminated by multiple genetically distinct lineages of bloodstream *Escherichia coli*. *Commun. Med.* 5:260. 10.1038/s43856-025-00972-x 40595425 PMC12216325

[B30] Suarez-Yana T. Salgado-Caxito M. Hayer J. Rojas-Sereno Z. E. Pino-Hurtado M. S. Campana-Burguet A. et al. (2024). ESBL-producing *Escherichia coli* prevalence and sharing across seabirds of central Chile. *Sci. Total Environ.* 951:175475. 10.1016/j.scitotenv.2024.175475 39142400

[B31] Sultan I. Siddiqui M. T. Gogry F. A. Haq Q. M. R. (2022). Molecular characterization of resistance determinants and mobile genetic elements of ESBL producing multidrug-resistant bacteria from freshwater lakes in Kashmir, India. *Sci. Total Environ.* 827:154221. 10.1016/j.scitotenv.2022.154221 35245551

[B32] Tian X. Zheng X. Sun Y. Fang R. Zhang S. Zhang X. et al. (2020). Molecular mechanisms and epidemiology of carbapenem-resistant *Escherichia coli* isolated from Chinese patients during 2002-2017. *Infect. Drug Resist.* 13 501–512. 10.2147/idr.S232010 32110061 PMC7035005

[B33] Walti C. S. Halpern A. B. Xie H. Kiem E. S. Chung E. L. Schonhoff K. G. et al. (2023). Infectious complications after intensive chemotherapy with CLAG-M versus 7+3 for AML and other high-grade myeloid neoplasms. *Leukemia* 37 298–307. 10.1038/s41375-022-01786-9 36509892

[B34] Wang Q. Hermannsson K. Masson E. Bergman P. Guethmundsson G. H. (2025). Host-directed therapies modulating innate immunity against infection in hematologic malignancies. *Blood Rev.* 70:101255. 10.1016/j.blre.2024.101255 39690006

[B35] Wang Y. Hu H. Shi Q. Zhang P. Zhao D. Jiang Y. et al. (2024). Prevalence and characteristics of ertapenem-mono-resistant isolates among carbapenem-resistant Enterobacterales in China. *Emerg. Microbes Infect.* 13:2332658. 10.1080/22221751.2024.2332658 38517707 PMC10993752

[B36] Wei D. W. Wong N. K. Song Y. Zhang G. Wang C. Li J. et al. (2021). IS26 veers genomic plasticity and genetic rearrangement toward carbapenem hyperresistance under sublethal antibiotics. *mBio* 13:e0334021. 10.1128/mbio.03340-21 35130728 PMC8822349

[B37] Wein T. Wang Y. Hülter N. F. Hammerschmidt K. Dagan T. (2020). Antibiotics interfere with the evolution of plasmid stability. *Curr. Biol.* 30 3841–3847.e4. 10.1016/j.cub.2020.07.019 32795438

[B38] Wirth T. Falush D. Lan R. Colles F. Mensa P. Wieler L. H. et al. (2006). Sex and virulence in *Escherichia coli*: An evolutionary perspective. *Mol. Microbiol.* 60 1136–1151. 10.1111/j.1365-2958.2006.05172.x 16689791 PMC1557465

[B39] Yang L. Shen Y. Jiang J. Wang X. Shao D. Lam M. M. C. et al. (2022). Distinct increase in antimicrobial resistance genes among *Escherichia coli* during 50 years of antimicrobial use in livestock production in China. *Nat. Food* 3 197–205. 10.1038/s43016-022-00470-6 37117646

[B40] Yao Z. C. Sun Y. Ma Z. X. Chen H. C. Fu Q. X. Xia P. et al. (2025). Longitudinal analysis of carbapenem-resistant *Escherichia coli* in a Southern Chinese hospital (2015-2021): Epidemiology, genetics, and resistance mechanisms. *Int. J. Antimicrob. Agents* 66:107577. 10.1016/j.ijantimicag.2025.107577 40692074

[B41] Yeoh D. K. Boast A. Wen S. C. Williams P. C. Voss L. Ritchie B. et al. (2025). Drug-resistant gram-negative bacterial infections in children in the Oceania region: Review of the epidemiology, antimicrobial availability, treatment, clinical trial and pharmacokinetic data, and key evidence gaps. *Lancet Reg. Health West. Pac.* 64:101735. 10.1016/j.lanwpc.2025.101735 41368010 PMC12685515

