# Supplementary material for: Comparative analysis of virulence and resistance gene profiles between carbapenem-resistant and ESBL-producing Escherichia coli
Source: Front Microbiol. 2026 Jul 15;17:1843914. doi: 10.3389/fmicb.2026.1843914 (PMC13416673; doi:10.3389/fmicb.2026.1843914)
Supplement: Supplementary file 1 [file Table_1.DOCX]

**Table 1**

**The sequences of primers for house-keeping genes used in this study.**

|  | Primer sequence (5’-3’) | Product size (bp) | ℃ | | |
| --- | --- | --- | --- | --- | --- |
| *adk* | F:5’-ATTCTGCTTGGCGCTCCGGG-3’ | 583 | | 54 |  |
|  | R:5’-CCGTCAACTTTCGCGTATTT-3’ |  | |  |  |
| *fumC* | R1:5’-TCCCGGCAGATAAGCTGTGG-3’  F:5’-TCACAGGTCGCCAGCGCTTC-3’ | 806 | | 54 |  |
|  | R:5’-GTACGCAGCGAAAAAGATTC-3’ |  | |  |  |
| *gyrB* | F:5’-TCGGCGACACGGATGACGGC-3’  R1:5’-GTCCATGTAGGCGTTCAGGG-3’ | 911 | | 60 |  |
|  | R:5’-ATCAGGCCTTCACGCGCATC-3’ |  | |  |  |
| *icd* | F:5’-ATGGAAAGTAAAGTAGTTGTTCCGGCACA-3’ | 878 | | 54 |  |
|  | R:5’-GGACGCAGCAGGATCTGTT-3’ |  | |  |  |
| *mdh* | F:5’-ATGAAAGTCGCAGTCCTCGGCGCTGCTGGCGG-3’ | 932 | | 60 |  |
|  | R:5’-TTAACGAACTCCTGCCCCAGAGCGATATCTTTCTT-3’  F1:5’-AGCGCGTTCTGTTCAAATGC-3’  R1:5’-CAGGTTCAGAACTCTCTCTGT-3’ |  | |  |  |
| *purA* | F1:5’-TCGGTAACGGTGTTGTGCTG-3’ | 816 | | 54 |  |
|  | F:5’-CGCGCTGATGAAAGAGATGA-3’  R:5’-CATACGGTAAGCCACGCAGA-3’ |  | |  |  |
| *recA* | R1:5’-AGCGTGAAGGTAAAACCTGTG-3’  F:5’-CGCATTCGCTTTACCCTGACC-3’ | 780 | | 58 |  |
|  | F1:5’-ACCTTTGTAGCTGTACCACG-3’  R:5’-TCGTCGAAATCTACGGACCGGA-3’ |  | |  |  |

(Wirth et al., 2006)

Wirth, T., Falush, D., Lan, R., Colles, F., Mensa, P., Wieler, L.H., et al. (2006). Sex and virulence in Escherichia coli: an evolutionary perspective. *Mol Microbiol* 60**,** 1136-1151. doi:10.1111/j.1365-2958.2006.05172.x.

**Table 2**

**The sequences of primers for virulence genes used in this study.**

|  | Primer sequence (5’-3’) | Product size (bp) | ℃ |
| --- | --- | --- | --- |
| *FimH* | F: TCGAGAACGGATAAGCCGTGG | 508 | 60 |
|  | R: GCAGTCACCTGCCCTCCGGTA |  |  |
| *PapA* | F: ATGGCAGTGGTGTCTTTTGGTG | 720 | 60 |
|  | R: CGTCCCACCATACGTGCTCTTC |  |  |
| *PapC* | F: GTGGCAGTATGAGTAATGACCGTTA | 200 | 60 |
|  | R: ATATCCTTTCTGCAGGGATGCAATA |  |  |
| *FocG* | F: CAGCACAGGCAGTGGATACGA | 364 | 61 |
|  | R: GAATGTCGCCTGCCCATTGCT |  |  |
| *sfa* | F: CTCCGGAGAACTGGGTGCATCTTAC | 410 | 60 |
|  | R:CGGAGGAGTAATTACAAACCTGGCA |  |  |
| *iroN* | F: AAGTCAAAGCAGGGGTTGCCCG | 667 | 62 |
|  | R: GACGCCGACATTAAGACGCAG |  |  |
| *FyuA* | F: TGATTAACCCCGCGACGGGAA | 880 | 60 |
|  | R: CGCAGTAGGCACGATGTTGTA |  |  |
| *iutA* | F: GGCTGGACATCATGGGAACTGG | 300 | 60 |
|  | R: CGTCGGGAACGGGTAGAATCG |  |  |
| *sitA* | F: AGGGGGCACAACTGATTCTCG | 608 | 60 |
|  | R: TACCGGGCCGTTTTCTGTGC |  |  |
| *hlyA* | F: AACAAGGATAAGCACTGTTCTGGCT | 1177 | 60 |
|  | R: ACCATATAAGCGGTCATTCCCGTCA |  |  |
| *cnf1* | F: AAGATGGAGTTTCCTATGCAGGAG | 498 | 60 |
|  | R: CATTCAGAGTCCTGCCCTCATTATT |  |  |
| *cvaC* | F: CACACACAAACGGGAGCTGTT | 680 | 60 |
|  | R: CTTCCCGCAGCATAGTTCCAT |  |  |
| *traT* | F: GGTGTGGTGCGATGAGCACAG | 290 | 60 |
|  | R: CACGGTTCAGCCATCCCTGAG |  |  |
| *kpsMT II* | F: GCGCATTTGCTGATACTGTTG | 272 | 56 |
|  | R: CATCCAGACGATAAGCATGAGCA |  |  |
| *kpsMT K5* | F: CAGTATCAGCAATCGTTCTGTA | 159 | 55 |
|  | R: CATCCAGACGATAAGCATGAGCA |  |  |
| *kpsMT K1* | F: TAGCAAACGTTCTATTGGTGC | 153 | 57 |
|  | R: CATCCAGACGATAAGCATGAGCA |  |  |
| *rfc* | F: ATCCATCAGGAGGGGACTGGA | 778 | 58 |
|  | R: AACCATACCAACCAATGCGAG |  |  |
| PAI | F: GGACATCCTGTTACAGCGCGCA | 930 | 63 |
|  | R: TCGCCACCAATCACAGCCGAAC |  |  |

(Johnson and Stell, 2000;Rodriguez-Siek et al., 2005)

Rodriguez-Siek, K.E., Giddings, C.W., Doetkott, C., Johnson, T.J., Fakhr, M.K., and Nolan, L.K. (2005). Comparison of Escherichia coli isolates implicated in human urinary tract infection and avian colibacillosis. *Microbiology (Reading)* 151**,** 2097-2110. doi:10.1099/mic.0.27499-0.

Rodriguez-Siek, K.E., Giddings, C.W., Doetkott, C., Johnson, T.J., Fakhr, M.K., and Nolan, L.K. (2005). Comparison of Escherichia coli isolates implicated in human urinary tract infection and avian colibacillosis. *Microbiology (Reading)* 151**,** 2097-2110. doi:10.1099/mic.0.27499-0.

**Table 3**

**The sequences of primers for resistance genes used in this study.**

|  | Primer sequence (5’-3’) | Product size (bp) | ℃ |
| --- | --- | --- | --- |

| *bla*_TEM_ | F: TCGGGGAAATGTGCG | 972 | 55 |
| --- | --- | --- | --- |
|  | R: TGCTTAATCAGTGAGGCACC |  |  |
| *bla*_SHV_ | F:GGTTATGCGTTATATTCGCC | 865 | 55 |
|  | R: TTAGCGTTGCCAGTGCTC |  |  |
| *bla_CTX-M_* | F:TACCGCAGATAATACGCAG | 355 | 55 |
|  | R:CAGCGTAGGTTCAGTGCGATC |  |  |
| *bla*_NDM_ | F: GGTTTGGCGATCTGGTTTTC | 621 | 53 |
|  | R: CGGAATGGCTCATCACGATC |  |  |
| *bla_KPC_* | F: TGTCACTGTATCGCCGTCTAG | 881 | 54 |
|  | R: GGTTTAAYAAAACAACCACC |  |  |
| *bla_IMP_* | F: GGAATAGAGTGGCTTAAYTCT | 232 | 52 |
|  | R: GGTTTAAYAAAACAACCACC |  |  |
| *bla*_VIM_ | F:ACTGGGCTACCTCTGCTTCA | 390 | 56 |
|  | R: CTTGCATGAGCCATCTTTCA |  |  |
| *bla*_OXA_ | F: GCGTGGTTAAGGATGAACAC | 438 | 58 |
|  | R: CATCAAGTTCAACCCAACCG |  |  |

*(Miao et al., 2018;Wang et al., 2020)*

Miao, M., Wen, H., Xu, P., Niu, S., Lv, J., Xie, X., et al. (2018). Genetic Diversity of Carbapenem-Resistant Enterobacteriaceae (CRE) Clinical Isolates From a Tertiary Hospital in Eastern China. *Front Microbiol* 9**,** 3341. doi:10.3389/fmicb.2018.03341.

Wang, B., Pan, F., Wang, C., Zhao, W., Sun, Y., Zhang, T., et al. (2020). Molecular epidemiology of Carbapenem-resistant Klebsiella pneumoniae in a paediatric hospital in China. *Int J Infect Dis* 93**,** 311-319. doi:10.1016/j.ijid.2020.02.009.
